# Amoxicillin-Induced Hypersensitivity After DRESS To Carbamazepine

**DOI:** 10.1097/WOX.0b013e3181eab930

**Published:** 2010-07-15

**Authors:** Karim Aouam, Ben Fredj Nadia, Chaabane Amel, Boughattas Naceur

**Affiliations:** 1 Service de Pharmacologie, Faculté de Médecine, Monastir, Tunisia

**Keywords:** hypersensitivity, amoxicillin, co-sensitization, DRESS, carbamazepine, skin tests

## Abstract

The anticonvulsant hypersensitivity syndrome, also known as drug rash eosinophilia and systemic symptoms (DRESS), is a rare but severe form of adverse cutaneous reaction. Several aromatic anticonvulsant drugs, such as carbamazepine (CBZ), phenytoin, or phenobarbital have been frequently associated with the onset of DRESS. Cross-reactivity among the aromatic anticonvulsants frequently occurs (40 to 80% of patients). However, cross reactivity with other drugs such as betalactams have exceptionally been reported. We report a clinical observation describing a DRESS associated with CBZ with a subsequent hypersensitivity to amoxicillin (AMX). A 34-year-old male with a 20-year history of epilepsy was treated with valproic acid and phenobarbital. As he had frequent convulsive fits, CBZ was added. Thirty-four days later, the patient developed hyperthermia (39.5°C), cervical lymphadenopathy, and generalized cutaneous exfoliated maculae and papulae. Biochemical investigation was characterized by a white cell count of (16.1 × 103/*μ*L, 17% eosinophils) and increased levels of aspartate aminotransferase and alanine aminotransferase (50 and 116 IU/L, respectively). CBZ was discontinued. One month later, all the symptoms were progressively relieved. Six weeks after complete recovery, prick and patch skin tests were performed. They were strongly positive at 48-hour reading. About 2 years later, the patient exhibited an extensive pruritic skin rash, 2 days after AMX intake. Laboratory exams showed eosinophilia (7%) but neither elevated liver enzymes nor renal dysfunction. All these symptoms have disappeared 5 days after AMX withdrawal. Intradermal test to AMX was positive but not to other betalactams. Throughout this clinical observation, we report a CBZ-induced DRESS and describe the possibility of cross reactivity between CBZ and AMX. This cross reactivity was observed despite the lack of chemical similarity between both drugs.

## 

Drug reaction with eosinophilia and systemic symptoms (DRESS) also known as hypersensitivity syndrome, is rare but one of the most severe forms of drug reaction. It associates fever, skin eruption, eosinophilia, and multiple organ involvement (lymph node enlargement, hepatitis, pneumonitis, renal dysfunction, and so forth) [1].

Aromatic anticonvulsant drugs [eg, phenytoin, phenobarbital, and carbamazepine (CBZ)] are still the principal drugs that elicit this syndrome. The latter has been attributed to a disorder in the metabolic patterns of the aromatic anticonvulsants leading to an excess of toxic metabolite [2]. Cross reactivity explained by a chemical or antigenic similitude has been well described between anticonvulsant drugs [3,4]. However, limited data have reported the development of drug hypersensitivity after a history of DRESS syndrome induced by other chemically unrelated drugs [5,6]. We report a case of amoxicillin (AMX) hypersensitivity after DRESS syndrome because of CBZ.

## Case Report

**Figure 1:**
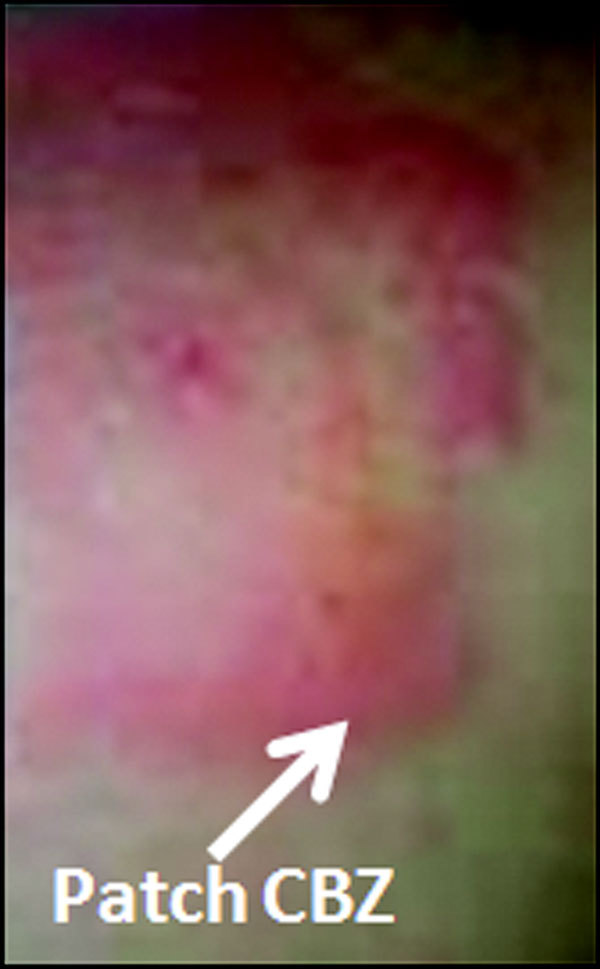
**Positive patch test to CBZ at 48-hour reading**.

A 34-year-old male with a 20-year history of epilepsy was treated with valproic acid (500 mg 3 times daily) and phenobarbital (200 mg once a day). As he had frequent convulsive fits, CBZ was added. Thirty-four days later, the patient developed hyperthermia and cervical lymphadenopathy. Initially, he was diagnosed with lymphadenitis and, thus, a therapy for AMX-clavulanic acid (1 g twice a day) and acetaminophen (500 mg 3 times a day), was started. Two days later, a generalized cutaneous eruption (exfoliated and confluent maculae and papulae associated with facial angioedema) was also observed. The laboratory findings showed an abnormal white cell count (16.1 × 10^3^/*μ*l, 17% eosinophils), a liver dysfunction with an aspartate aminotransferase (AST) level of 50 IU/L and an alanine AST level (ALT) of 116 IU/L (normal 4-40 IU/L), a lactate deshydrogenase level of 3197 (normal 190-430 IU/L). The platelet count, INR, the serum levels of immunoglobulins, and the renal function were conversely normal and no atypical lymphocytes were found. The thoracic imaging did not show any abnormalities. The serologic test of Human Herpes Virus 6 (HHV6) was positive (IgM anti-HHV6 detected). Tests for other viral infections including cytomegalovirus, Epstein-Barr virus, hepatitis B and C, and HIV were all negative. CBZ, AMX-clavulanic acid, and acetaminophen were then discontinued and cetirizine (10 mg once a day) was administered. About 1 month later, the skin eruption, fever, lymphadenopathy, liver dysfunction, and eosinophilia progressively disappeared. Six weeks after complete recovery, a patch test to CBZ was performed and was strongly positive at 48-hour reading (Figure 1).

**Figure 2:**
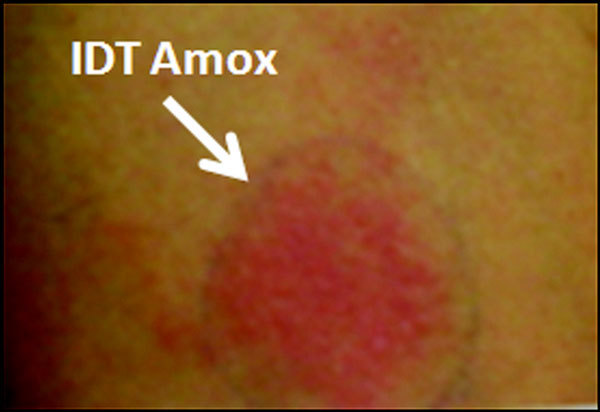
**Positive intradermal test to AMX at 48-hour reading**.

About 2 years later, the patient was treated with AMX (2 g daily) for a dental abutment. On day 2 of this treatment, he presented a generalized maculo-papular eruption with neither fever nor lymph node swelling. The laboratory findings showed eosinophilia (white cell count: 8.1 × 10^3^/*μ*l, 7% eosinophils) with normal hepatic and renal function. AMX was discontinued with favorable course 1 week later. Two months later, an intradermal test to AMX (20 mg/ml) was performed, and was positive at 48-hour reading (Figure 2). An intradermal test to penicillin G (20 000 IU/ml), cefazolin (20 mg/ml), and cefotaxime (20 mg/ml) was conversely negative (Figure 2).

## Discussion

We describe a case of hypersensitivity to AMX after a DRESS induced by CBZ. We believe that DRESS would obviously be related to CBZ in view of the after arguments: 1) a clear temporal relationship between the administration of CBZ and the symptoms onset (5 and 10 weeks, respectively, typically 1 to 12 weeks [7,8]); 2) remission of the symptomatological pattern after withdrawal of this drug; 3) the association of different symptoms evoking a clinical picture of DRESS syndrome; and 4) a positive patch test to CBZ. Regarding the subsequent episode of hypersensitivity in our patient, the responsibility of AMX was established according to the clear temporal relationship between AMX intake and the reaction onset, the remission of the symptoms after AMX withdrawal and the positive intradermal test to AMX.

DRESS is a nosological entity characterized mainly by a potentially life-threatening drug-induced cutaneous eruption. Several drugs including anticonvulsant agents, sulfonamides, and allopurinol, have been associated with an increased risk of inducing such a syndrome. The clinical picture of DRESS includes fever, morbiliform eruption, lymphadenopathy, pleural effusion, liver and renal dysfunctions, hematological abnormalities such as eosinophilia, and atypical lymphocytosis [9]. Our patient did not exhibit any renal or respiratory disorders (creatinine and thoracic imaging were normal).

According to the results of some studies,[10,11] it seems that several mechanisms must simultaneously occur to produce the massive specific and nonspecific T cell activity that is a characteristic feature of DRESS. Enough exposure time to a drug able to form chemically active toxic metabolites is required (or, alternatively, the native drug or its metabolites must be exposed on MHCII-matched antigen presenting cells to specific cytotoxic T cells). It has been suggested that a defect in the epoxide hydroxylases, which induces the accumulation of CBZ toxic metabolites, may lead to cell death or contribute to the formation of antigens triggering the immune reaction involved in DRESS [2]. However, in our patient because the patch test was positive to CBZ, it can be argued that the drug reaction may be caused by CBZ itself and not its reactive metabolite. It is reported that a reactivation of a latent HHV-6 infection during a period of transient immunosuppression would stimulate a massive expansion of HHV6-specific and -nonspecific bystander CD8^+ ^and CD4^+ ^T cells and cause full development of DRESS symptoms. However, it is not completely clear whether HHV6 reactivation is a consequence of a strong immune modulation during DRESS or a cofactor that favors the manifestation of this syndrome [12]. Anyway, we did not notice a reactivation of HHV6 in our patient because the serological test showed a primary infection. As our patient exhibited a hypersensitivity reaction to AMX after a DRESS episode, it can be argued that a possible AMX-CBZ cosensitization has occurred. AMX had been administered previously during the episode of DRESS in our patient. We had a positive intradermal test to AMX with 48 hours reading, denoting a delayed hypersensitivity to this drug. The intradermal test to other betalactams was negative, revealing a lack of cross reactivity between AMX and these drugs. The hypersensitivity reaction that occurred in our patient was mild compared with the initial one. Indeed, we noticed a skin rash and eosinophilia with no organ involvement. Cross reactivity between anticonvulsant drugs has been described widely in the literature when the initial reaction is DRESS. It is known to be as high as 80% among aromatic ones [4]. We have recently described a cross reactivity between CBZ and lamotrigine, aromatic and non-aromatic anticonvulsants, respectively [13]. To our knowledge, 5 clinical observations describing a sensitization to chemically different drugs taken during a previous episode of anticonvulsant-induced DRESS have been reported so far [5,6]. Only 2 cases reported a possible AMX-CBZ cosensitization. The first described a generalized rash and facial angioedema occurring 7 hours after taking AMX in a patient who took this drug during a previous CBZ-related DRESS. The second refers to a 68-year-old woman with a history of CBZ-induced DRESS who had a positive patch test to AMX. In this respect, some authors have suggested that the supposed cross-reactions between such drugs, without any chemical or antigenic similitude, are because of the fact that the drug responsible for the second reaction was administered during the immunologic depression occurring during a first DRESS episode [14]. Our clinical observation appears to support this hypothesis. In view of these considerations, it has been suggested that DRESS episode may elicit a massive nonspecific activation of the immune system, which will provide the enhanced expression of costimulatory molecules and proinflammatory cytokines. The latter will allow a more efficient presentation of chemical antigens to antigen-presenting cells and consequently decrease the level of tolerance to drugs,[5] in particular those known to be potentially immunogenic, such as AMX antibiotics whether the drug was taken or not during the initial phase of DRESS.

## Conclusion

Throughout this report, we point out a possible cosensitization to chemically or antigenically unrelated drugs, CBZ on the one hand, and AMX, on the other hand. Thus, clinicians should be aware when prescribing AMX to a patient with a previous history of DRESS syndrome. Skin tests to betalactams should be performed in such a patient to identify whether he could or not tolerate these drugs.
